# Requirements for Differentiation of an Immature CD4^+^8^+^ T-Cell Line

**DOI:** 10.1155/1997/26547

**Published:** 1997

**Authors:** Jenefer Dekoning, Jonathan G. Kaye

**Affiliations:** Department of lmmunology The Scripps Research Institute 10550 North Torrey Pines Road La Jolla California 92037 USA

**Keywords:** Cellular differentiation, T lymphocytes, T-cell receptors, signaling molecules, cell signaling

## Abstract

The CD3ɛ and ζ chains of the TCR have been shown to possess independent signaling capabilities. Studies with chimeric molecules containing the cytoplasmic domains of either ζ or ɛ have suggested that these two structurally distinct members of the TCR-CD3 complex are able to function autonomously and have redundant features in the context of TCR-signal transduction in mature T cells. Expression of a chimeric human IL-2-receptor-ζ-chain molecule in the CD4^+^8^+^ T-cell line, DPK, has enabled us to directly analyze responses initiated by the ζ-chain-signaling module alone within the context of immature T-cell differentiation. In this paper, we show that antibody crosslinking of the chimeric ζ chain delivers only a limited activation signal as measured by Ca[2^+^] flux, induction of low-level CD5 expression, and minimal differentiation as assessed by loss of cell-surface CD8 expression. TCR-induced activation through antibody crosslinking of the endogenous CD3ɛ receptor in the absence of costimulation was also relatively inefficient in initiating activation and differentiation. However, co-crosslinking of the CD4 coreceptor with CD3 resulted in a synergistic response, where as there was little effect of co-crosslinking of CD4 and the ζ-chain chimera. Striking differences were also observed in the substrate pattern of tyrosine phosphorylation, as well as lymphokine secretion following triggering through the intact TCR versus the ζ chain alone. These results indicate that although the ζ-chain may possess some signaling capacities similar to that of the intact TCR, it appears to have limited function as an autonomous subunit in initiating CD4^+^8^+^ T-cell differentiation.


*Developmental Immunology*, 1997, Vol. 5, pp. 91–103
Reprints available directly from the publisher
Photocopying permitted by license only

© 1997 OPA (Overseas Publishers Association)
Amsterdam B.V. Published under license in The Netherlands
under the Harwood Academic Publishers imprint
part of The Gordon and Breach Publishing Group
Printed in Malaysia

### Requirements for Differentiation of an Immature $CD4^+8^+$ T-Cell Line

JENEFER DEKONING and JONATHAN G. KAYE*

*Department of Immunology, The Scripps Research Institute, 10550 North Torrey Pines Road, La Jolla, California 92037*

*(Received 28 September 1996)*

The CD3ε and ζ chains of the TCR have been shown to possess independent signaling capabilities. Studies with chimeric molecules containing the cytoplasmic domains of either ζ or ε have suggested that these two structurally distinct members of the TCR-CD3 complex are able to function autonomously and have redundant features in the context of TCR-signal transduction in mature T cells. Expression of a chimeric human IL-2-receptor-ζ-chain molecule in the $CD4^+8^+$ T-cell line, DPK, has enabled us to directly analyze responses initiated by the ζ-chain-signaling module alone within the context of immature T-cell differentiation. In this paper, we show that antibody crosslinking of the chimeric ζ chain delivers only a limited activation signal as measured by $Ca[2^+]$ flux, induction of low-level CD5 expression, and minimal differentiation as assessed by loss of cell-surface CD8 expression. TCR-induced activation through antibody crosslinking of the endogenous CD3ε receptor in the absence of costimulation was also relatively inefficient in initiating activation and differentiation. However, co-crosslinking of the CD4 coreceptor with CD3 resulted in a synergistic response, where as there was little effect of co-crosslinking of CD4 and the ζ-chain chimera. Striking differences were also observed in the substrate pattern of tyrosine phosphorylation, as well as lymphokine secretion following triggering through the intact TCR versus the ζ chain alone. These results indicate that although the ζ-chain may possess some signaling capacities similar to that of the intact TCR, it appears to have limited function as an autonomous subunit in initiating $CD4^+8^+$ T-cell differentiation.

*Keywords:* Cellular differentiation, T lymphocytes, T-cell receptors, signaling molecules, cell signaling

### INTRODUCTION

The T-cell receptor (TCR)-CD3 complex is comprised of an antigen-specific α- and β-chain heterodimer associated with the invariant chains, γ, δ, ε, ζ, and η. Together these proteins are responsible for the link between recognition and the intracellular signaling pathways that initiate cellular activation. Separately, the clonotypic TCR α and β antigen-receptor chains do not themselves possess intrinsic signaling capabilities, but rely on coupling to one or more members of the CD3 complex (Weiss, 1991). Within this multimeric complex, both the CD3ε and the ζ chains have been shown to be independently capable of transducing signals for T-cell activation (Irving and Weiss, 1991; Letourneur and Klausner, 1991; Wegener et al., 1992). Thus, it has

*Corresponding author.

been proposed that the TCR is composed of two autonomous signaling modules consisting of γδε and ζ–ζ chain subunits. The signaling function of ε and ζ chains resides in their cytoplasmic domains, which contain one or more conserved peptide sequences called immunoreceptor tyrosine-based activation motifs (ITAM) (Weissman et al., 1988; Reth, 1989; Flaswinkel et al., 1995). Mutational analyses have demonstrated the importance of these tyrosine-based motifs in conferring signaling capability (Letourneur and Klausner, 1992).

Analysis of the role of the CD3ε and ζ chains in TCR-mediated signaling has come largely from studies of chimeric molecules expressed in various T-cell hybridomas or tumors (Irving and Weiss, 1991; Romeo and Seed, 1991; Letourneur and Klausner, 1992; Wegener et al., 1992). Such chimeric molecules are comprised of the cytoplasmic domains of ε or ζ fused to extracellular and transmembrane domains of unrelated proteins. Antibody-mediated crosslinking of these chimeric proteins results in intracellular activation events similar to those generated by the endogenous TCR. These include proximal signaling such as activation of protein tyrosine kinases and Ca[$2^+$] mobilization (Irving and Weiss, 1991; Letourneur and Klausner, 1992), and distal activation events such as CD69 expression (Irving and Weiss, 1991), and IL-2 production (Irving and Weiss, 1991; Letourneur and Klausner, 1991, 1992; Wegener, et al., 1992). Although most studies have analyzed TCR signaling in the context of transformed T-cell lines, a recent study analyzed the function of chimeric molecules expressed in transgenic mice (Brocker and Karjalainen, 1995). In this instance, a ζ-chain chimeric molecule was found to be functional only in activated T cells but not in resting T lymphocytes. Thus, the requirements for initiating activation of naive cells may be more stringent than that observed in T-cell hybridomas.

Evidence that the ζ chain plays a critical role in T-cell development in addition to mature T-cell activation is primarily from studies of both CD3ζ-deficient mice and CD3ζ-deficient mice that express various mutated forms of a ζ-chain transgene (Sussman et al., 1988; Liu et al., 1993; Love et al., 1993; Malissen et al., 1993; Ohno et al., 1993; Love et al., 1994; Shores et al., 1994; Shinkai et al., 1995). These studies demonstrated that although the ζ chain is required for efficient surface expression of TCR complexes, signals delivered via the ζ-chain cytoplasmic domain are not obligatory for positive selection. Thus, maturation from the double-positive ($CD4^+8^+$) to the single-positive thymocyte stage proceeds with TCRs that express a truncated ζ-chain-lacking cytoplasmic-domain ITAMs, although less efficiently (Shores et al., 1994). Whether signals from the ζ chain are redundant with those from the ε chain in this instance, whether there are compensatory mechanisms, or whether ζ-chain-generated signals play little role in positive selection is not clear. Crosslinking of a ζ-chain chimeric protein on transgene-expressing $CD4^+8^+$ thymocytes demonstrated that ζ-chain signals alone were sufficient to elicit programmed cell death in immature thymocytes (Shinkai et al., 1995). However, positive selection could not be addressed in this transgenic model due to the intrinsic property of double-positive thymocytes to succumb to apoptotic cell death on crosslinking of the TCR (Shi et al., 1989; Smith et al., 1989).

In order to further examine the role of the ζ chain in positive selection, we have taken advantage of an *in vitro* model of positive selection we have established. The immature double-positive thymocyte cell line DPK was derived from a TCR transgenic mouse that expresses a Vα11/Vβ3 TCR specific for pigeon cytochrome c peptide (PC) and $E^k$ class II MHC molecule (Kaye and Ellenberger, 1992). On stimulation *in vitro* or *in vivo*, DPK cells undergo a differentiation process similar to normal thymocytes undergoing positive selection (Kaye and Ellenberger, 1992; Poirier et al., 1994; DeKoning et al., 1995). Although DPK cells are identical in many ways to immature thymocytes, they have been previously shown to be specifically resistant to antigen- or anti-TCR/CD3-induced apoptosis (Kaye and Ellenberger, 1992). Rather, we have shown that DPK cells can be triggered to differentiate in response to the anti-CD3ε antibody in the presence of costimulation (DeKoning et al., 1995). We have taken advantage of this fact in order to assess the role of the ζ chain in positive selection. The TTζ chimeric molecule (Letourneur and Klausner, 1991), composed of the extracellular and transmembrane domains of the human interleukin-2 receptor and the ζ-chain cytoplasmic domain, was expressed in DPK cells. This system has allowed us to examine the requirements for T-cell differentiation in an immature T cell induced by crosslinking of either the endogenous TCR or the ζ chain alone. We demonstrate in this paper that both early and late activation signals delivered by crosslinking of the ζ chain at the $CD4^{+}8^{+}$ T-cell stage appear to be qualitatively and/or quantitatively different than the activation signals induced by engagement of the endogenous TCR. These data indicate that, unlike induction of apoptosis in double positive thymocytes (Shinkai, et al., 1995), positive selection requires specific signals other than those mediated solely through the TCR ζ chain, even in conjunction with coreceptor cross-linking.

### RESULTS

### Requirements for Differentiation of an Immature T-Cell Line

The $CD4^{+}8^{+}$ DPK T-cell line differentiates into $CD4^{+}8^{-}$ cells on activation by pigeon cytochrome c peptide (PC) and $E^{k}$-bearing antigen-presenting cells. Coincident with downregulation of CD8, there are other cell-surface changes that occur during DPK cell differentiation that are also characteristic of thymocyte positive selection (Kaye and Ellenberger, 1992). However, unlike immature thymocytes, antibody crosslinking of CD3ε on DPK cells does not induce cell death. This fact allowed us to dissect out the requirements for TCR/CD3-mediated differentiation in this system.

In the absence of other costimulatory signals, plate-bound anti-CD3ε antibody is a poor activator of DPK differentiation, as indicated by the near absence of cells exhibiting a $CD8^{lo/-}$ phenotype, even when high concentrations of antibody are used (Figs. 1A and 1B). CD5 is normally expressed on thymocytes and mature T cells and is upregulated as a result of TCR engagement in double-positive thymocytes (Kearse et al., 1995; Tarakhovsky et al., 1995). Although DPK cells do not express detectable levels of CD5, it is upregulated during their differentiation. It is unclear why the basal level of CD5 is higher on normal double-positive thymocytes than DPK cells. However, the level of CD5 is also unusually low on H-Y transgenic double-positive thymocytes in a β2-microglobulin-deficient background (Dutz et al., 1995), suggesting that weak TCR interactions in the thymic environment may be required to maintain normal CD5 expression. In any case, CD5 induction remains a useful marker of DPK-cell differentiation as a consequence of TCR activation. In contrast to the failure of anti-CD3ε mAb to induce the loss of cell-surface CD8, high concentrations of anti-CD3ε mAb can induce surface expression of CD5 on the majority of DPK cells (Fig. 1B). These data indicate that the signals provided by anti-CD3ε can activate DPK cells, but do not efficiently initiate differentiation to a single-positive cell.

The results with anti-CD3ε mAb are in contrast to those obtained by activation with specific peptide antigen, a potent inducer of DPK-cell differentiation. One obvious difference in the antigen/MHC versus anti-CD3ε response of DPK cells is that the former would be expected to engage the CD4 coreceptor. In order to determine if the response to anti-CD3ε mAb could be affected by coreceptor engagement, anti-CD3ε and anti-CD4 mAbs were coimmobilized on a plate prior to incubation with DPK. As shown in Figs. 1A and 1B, the presence of anti-CD4 mAb dramatically increases the ability of DPK cells to differentiate as indicated by loss of cell-surface CD8 and induction of CD5. In order to determine whether the observed enhancement of the anti-CD3ε response by coreceptor engagement was specific, the effect of mAbs to LFA-1 and Thy-1 was tested. Both LFA-1 and Thy-1 are highly expressed by normal murine thymocytes and DPK cells (Kaye and Ellenberger, 1992; DeKoning et al., 1995). Neither addition of anti-LFA-1 nor anti-Thy-1 mAb resulted in an enhanced response to anti-CD3ε mAb (Fig. 1C). This suggests an active role for CD4 in DPK-cell differentiation rather than acting as a passive enhancer of adhesion.

### Expression of a hIL-2R TCRζ-Chain Chimeric Molecule in DPK Cells

In order to assess the potential of the ζ chain to contribute to either the activation or differentiation of T cells at the double-positive stage of development, we expressed the TTζ chimeric molecule in DPK cells by retroviral-mediated gene transfer. This chimeric molecule was constructed by exchanging the cytoplasmic

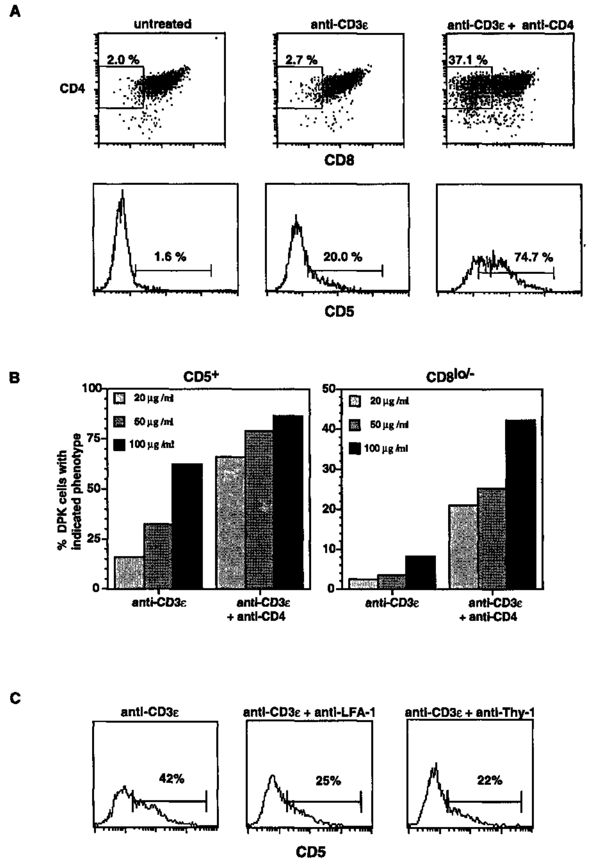

FIGURE 1 Stimulation of DPK cells by plate-bound anti-CD3ε 2C11 mAb is significantly enhanced by coimmobilization with anti-CD4 mAb. (A) DPK cells were incubated in either untreated wells or wells previously coated with 20 µg/ml 2C11 mAb in the presence or absence of 10 µg/ml anti-CD4 mAb. Cells were harvested after 3 days of culture and three-color stained for CD5, CD4, and CD8 and analyzed by flow cytometry. (B) DPK cells were incubated in wells previously coated with concentrations of 2C11 mAb, as indicated, in the presence or absence of anti-CD4 mAb, and analyzed by flow cytometry as in (A). Shown is the percentage of cells that were CD5 + or $CD8^{lo/}$ − gated, as in (A). (C) DPK cells were incubated in wells previously coated with 2C11 mAb alone or coimmobilized with anti-LFA-1 or anti-Thy-1 mAb. Cells were harvested after 3 days of culture and analyzed as described above.

tail of the murine ζ chain for the cytoplasmic tail of the human interleukin-2 receptor α-chain (TTζ) (Letourneur and Klausner, 1991). Cells were sorted for high-level expression of the chimeric molecule by flow cytometry employing a monoclonal antibody specific for the hIL-2R (anti-Tac). Figure 2 shows the level of cell-surface expression of the chimeric protein on DPK cells after FACS sorting. Cells were also sorted for low-level expression of the TTζ-chain and assayed comparatively for function (data not shown). However, as previously reported in studies employing transfected T-cell hybridomas (Letourneur and Klausner, 1992; Brocker and Karjalainen, 1995), the level of expression or overexpression of the chimera yielded qualitatively similar responses on stimulation. Transfected cells maintaining a high level of expression of the chimeric protein were thus called ZPDPK and used for all subsequent assays.

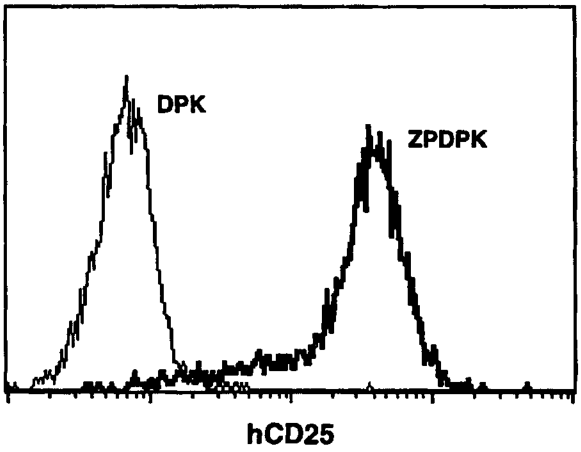

FIGURE 2 Cell-surface expression of the TTζ chimeric protein on DPK cells. Untransfected (grey) DPK and transfected (black) ZPDPK sorted for high expression of the hIL-2R receptor were stained with an anti-Tac antibody (hCD25), followed by a FITC-conjugated anti-mouse immunoglobulin secondary antibody.

### Early Activation Signals Delivered by Crosslinking of the TTζ-Chain Chimera

T-cell activation triggered either by antigen or TCR/CD3 antibodies results in tyrosine phosphorylation of intracellular substrates, including CD3ε and ζ, and subsequent $Ca[2^+]$ mobilization (Danielian et al., 1992; Tsygankov et al., 1992; Burkhardt et al., 1994; Chu and Littman, 1994). In studies employing transfected T-cell hybridomas, it has been shown that signaling through the ζ chain results in these early activation events (Irving and Weiss, 1991; Romeo and Seed, 1991; Letourneur and Klausner, 1992; Aoe et al., 1994). Additionally, phosphorylation of PTKs and phospholipase C induced by crosslinking of the CD3ε receptor have been shown to be enhanced by anti-CD4 co-crosslinking (Ledbetter et al., 1990; Chu and

Littman, 1994). However, although co-crosslinking of CD4 with the TCR has been demonstrated to potentiate the magnitude of resulting tyrosine phosphorylation in mature T cells, there does not appear to be a significant difference in the pattern of substrates phosphorylated as compared to CD3 crosslinking alone (Chu and Littman, 1994). Our analysis of tyrosine phosphorylation stimulated under comparable conditions in T cells at the CD4$^+$8$^+$ stage yielded similar results. ZPDPK cells were incubated with beads coated with anti-CD3$\varepsilon$ mAb in the presence or absence of anti-CD4 mAb. Anti-CD3$\varepsilon$ mAb stimulation can be seen to induce tyrosine phosphorylation of multiple substrates as compared to unactivated DPK cells (Fig. 3, lanes 1 and 2). On addition of anti-CD4 mAb, there was no difference in the magnitude of the response, nor were there apparent differences in the substrate pattern of phosphorylation from that of anti-CD3$\varepsilon$ mAb stimulation alone (Fig. 3, lanes 2 and 3). When these same cells were stimulated with anti-Tac mAb, there was a decrease (45 and 112kD) or loss (47, 50, and >112 kD) of tyrosine phosphorylation of some substrates, and little change in others (95 kD), as compared to anti-CD3$\varepsilon$ mAb activated cells (Fig. 3, lanes 2 and 4). Again, costimulation with anti-CD4 mAb did not markedly affect either the number of tyrosine phosphorylated proteins observed or the degree of phosphorylation (Fig. 3, lanes 4 and 5). These results suggest that although crosslinking of either the endogenous CD3 complex or the TT$\zeta$ chain can activate PTKs, there are quantitative and qualitative differences in the phosphorylation of cellular substrates induced by these two means of activation.

Following TCR-driven PTK activation, subsequent PLC$\gamma$1 activation leads to an increase in intracellular calcium levels (Gelfand, et al., 1988; Goldsmith and Weiss, 1988). As another measure of proximal signaling, the transfected ZPDPK cells were assayed for levels of intracellular Ca[2$^+$] after crosslinking of the respective TCR chains. Figure 4 demonstrates that following crosslinking, both endogenous CD3 and the chimeric TT$\zeta$ chain are capable of delivering the proximal signals necessary to induce a Ca[2$^+$] flux, although stimulation via anti-CD3$\varepsilon$ elicited a greater number of responding cells. These data suggest that both the intact TCR and isolated $\zeta$ chains can deliver overlapping but distinct early signals to the immature T cell.

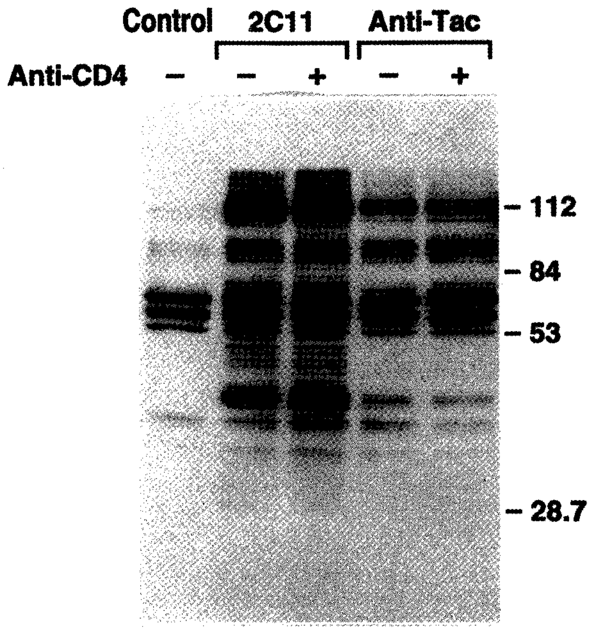

FIGURE 3 Crosslinking of either CD3 or TT$\zeta$ induces different substrate patterns of tyrosine phosphorylation. Transfected ZPDPK cells were incubated with rat-immunoglobulin coated beads (lane 1), 2C11 mAb coated beads (lane 2), 2C11 and anti-CD4 mAb coated beads (lane 3), anti-Tac mAb coated beads (lane 4), or anti-Tac and anti-CD4 mAb coated beads (lane 5) at 37°C for 30 min. Cells were lysed and equivalent volumes of whole cell lysates were resolved by Western blots probed with anti-phosphotyrosine mAb 4G10 and developed using enhanced chemiluminescence.

### The TT$\zeta$ Chain is a Weak Mediator of Induction for Late T-Cell Activation Events

Although TCR/CD3-mediated signaling leads to the rapid induction of PTKs and increased levels of intracellular Ca[2$^+$], these events may occur transiently and not lead to full activation as measured by downstream events such as lymphokine secretion. For example, it has been suggested that the length or persistence of the signal and whether a sustained Ca[2$^+$] increase occurs may be critical to the decision as to whether IL-2 gene activation is induced (Gelfand, et al., 1988; Goldsmith and Weiss, 1988). In order to address this issue, lymphokine production was analyzed in ZPDPK cells.

Undifferentiated DPK cells have been previously shown to secrete low levels of interleukin-2 (IL-2) and undetectable levels of interleukin-4 (IL-4) in response to antigen or plate-bound anti-CD3$\varepsilon$ mAb (Kaye and Ellenberger, 1992). However, activation of differentiated

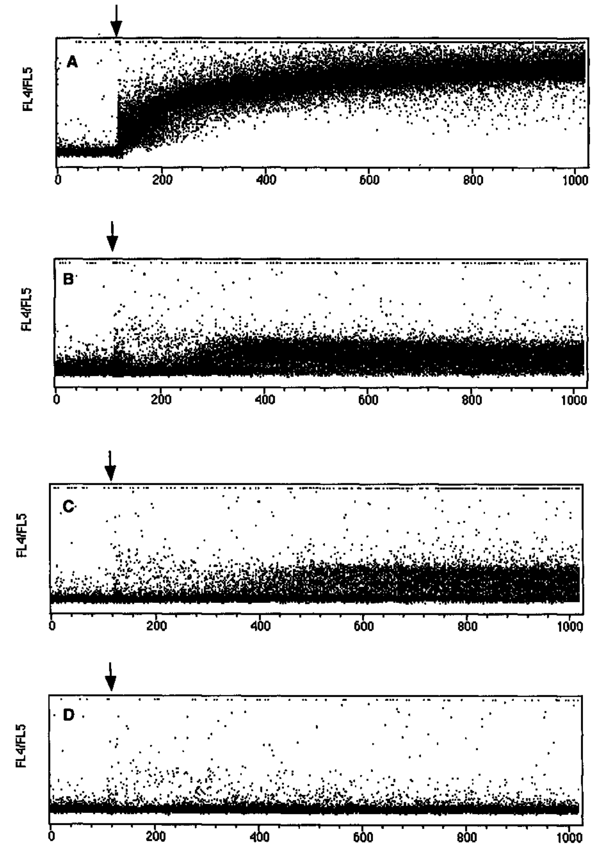

FIGURE 4 Crosslinking of the TTζ chimera results in an intracellular increase in [$Ca^{2+}$]. Changes in fluorescence over time were monitored in Indo-1-loaded ZPDPK cells stimulated either by (A) 25μg/ml ionomycin, (B) anti-CD3ε mAb stained cells crosslinked with a rabbit anti-hamster Ig secondary antibody, (C) anti-Tac mAb stained cells crosslinked with an anti-rat Ig secondary antibody, or (D) anti-rat Ig secondary antibody alone. The arrow indicates the time of addition of ionomycin or secondary cross linking antibody.

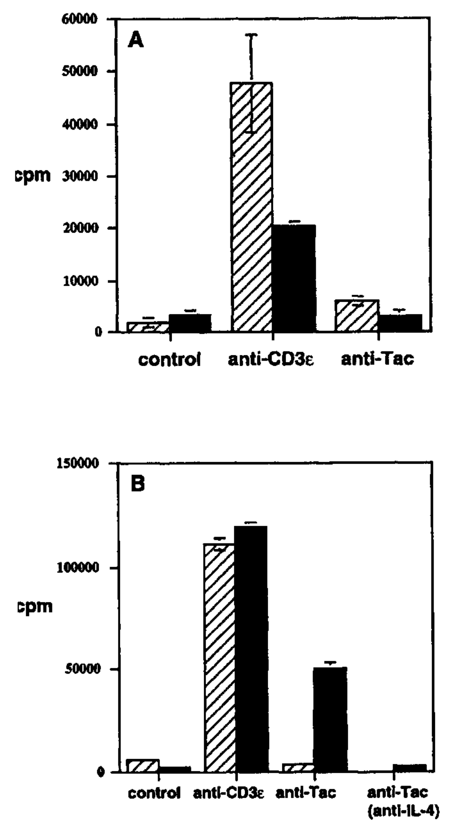

FIGURE 5 TTζ chimera mediated signaling is inefficient at inducing lymphokine secretion as compared to CD3-mediated signaling. (A) DPK (stippled bars) or ZPDPK (black bars) cells were incubated with plate-bound anti-CD3ε or anti-Tac mAb overnight. After 24 h of culture, supernatants were harvested and assayed for IL-2/IL-4 in a bioassay, as described in Materials and Methods. (B) DPK and ZPDPK cells were harvested after stimulation with 1 μM PC and DCEK-ICAM antigen presenting cells for 4 days, washed, and restimulated with plate-bound anti-CD3ε or anti-Tac mAb as above. Supernatants were harvested and assayed in a IL-2/IL-4 bioassay in the presence (anti-Tac stimulated ZPDPK only) or absence of anti-IL-4 blocking antibody as indicated.

DPK cells results in significant IL-4 production. When transfected ZPDPK cells were assayed for lymphokine production, a similar pattern was observed. In their undifferentiated state, stimulation of DPK or ZPDPK cells with anti-CD3ε mAb results in secretion of detectable levels of IL-2 (Fig. 5A). In contrast, DPK or ZPDPK cells harvested on day 4 of differentiation induced by coculture with antigen and antigen-presenting cells were found to secrete IL-4 in response to plate-bound anti-CD3ε mAb (Kaye and Ellenberger, 1992; Fig. 5). In order to determine if crosslinking of the TTζ-chain chimeric protein could deliver similar signals, undifferentiated or differentiated ZPDPK cells were stimulated by plate-bound anti-Tac antibody and assayed for production of IL-2/IL-4. Undifferentiated ZPDPK cells stimulated with anti-Tac antibody did not produce detectable levels of IL-2 or IL-4 (Fig. 5A). In contrast, differentiated ZPDPK cells harvested 4 days following activation by antigen produced detectable levels of IL-4 following crosslinking of the TTζ-chain chimeric protein (Fig. 5B). The level of IL-4 produced in response to anti-Tac, however, was significantly lower than the response typically observed with anti-CD3ε mAb. These results suggest that the outcome of signaling through the TTζ chain may depend on the differentiation state of the cell. In addition, TTζ signals may not be as efficient at inducing lymphokine gene activation as signals delivered via the intact TCR in this double-positive cell.

### Limited Differentiation in ZPDPK Cells on Crosslinking of the TTζ Chain

As crosslinking of the TTζ-chain chimera in ZPDPK cells did induce some proximal signaling events, it was of interest to determine if the chimeric protein could mediate differentiation. As observed with DPK cells, CD4 coengagement under conditions of limited anti-CD3ε antibody-mediated stimulation results in significantly enhanced differentiation in ZPDPK cells (Fig 6). In the absence of costimulation, when ZPDPK cells are triggered by incubation with plate-bound anti-Tac mAb, at an equivalent concentration to that of anti-CD3ε mAb, only a small percentage of cells are induced to differentiate as measured by downregulation of CD8 and upregulation of CD5. Most strikingly, co-crosslinking of Tac and the CD4 coreceptor had only a modest effect on CD5 expression, in sharp contrast to the results obtained by coengagement of CD3ε and CD4 (Figs. 1 and 6). Additionally, while holding anti-CD4 antibody concentrations constant, increasing the concentration of plate-bound 2C11 antibody led to a dose-dependent upregulation of CD5 expression (Fig. 1B). In contrast, increasing concentrations of anti-Tac mAb in combination with anti-CD4 did not enhance the response (data not shown).

### DISCUSSION

In defining the signaling requirements for the double-positive to single-positive transition of DPK cells, we found that anti-CD3ε mAb is relatively inefficient in mediating this differentiation. Instead, DPK cells activated via TCR crosslinking in the absence of any other costimulatory signals exhibit only minimal cell-surface CD8 loss, and low-level induction of CD69 (DeKoning et al., 1995) and CD5, as compared to that elicited in response to stimulation with specific antigen (Kaye and Ellenberger, 1992). The addition of a costimulatory signal, such as co-crosslinking of the TCR with the CD4 coreceptor, significantly enhanced differentiation, whereas anti-CD3ε mAb antibody in conjunction with antibodies against other highly expressed cell-surface proteins did not exhibit this effect. This was not too surprising given the critical role of the CD4 coreceptor in positive selection (Rahemtulla et al., 1991) and the synergistic effects on T-cell activation following co-crosslinking of the TCR/CD3 and CD4 coreceptor (reviewed in Miceli and Parnes, 1993; Chu and Littman, 1994; Baldari et al., 1995). We have also seen enhancement of antigen and anti-CD3 activation by ICAM-1 in this system (Kaye and Ellenberger, 1992; DeKoning et al., 1995), suggesting that there may be a number of pathways that can amplify TCR-generated signals and initiate differentiation.

The ζ chain has been demonstrated to be a critical component of TCR-mediated signaling, and both proximal and distal signals can be induced in T-cell hybridomas by stimulation through the ζ-chain cytoplasmic domain. However, the specific role of this signaling module in the differentiation of immature T cells has been more difficult to address, as crosslinking of either the endogenous TCR or chimeric proteins that possess the cytoplasmic domains of ζ or ε on double-positive thymocytes, results in apoptosis (Shi et al., 1989; Smith et al., 1989; Shinkai et al., 1995). DPK, cells in contrast, do not die in response to TCR engagement, but rather differentiate in response to specific antigen, thymic epithelial cells or anti-CD3ε crosslinking (Kaye and Ellenberger, 1992; Poirier et al., 1994; DeKoning et al., 1995). By expression of the TTζ chimeric protein in DPK cells, we were able to compare responses elicited from engagement of the endogenous TCR with those that result from crosslinking of the ζ chain in isolation, within the context of a double-positive T cell. Our results indicate that although engagement of the chimeric protein can mediate some signaling events, it is in general a poor activator of both proximal signaling and differentiation in this cell, even in the presence of coreceptor engagement. These results suggest that the ζ chain may have limited function in positive selection. It is also possible

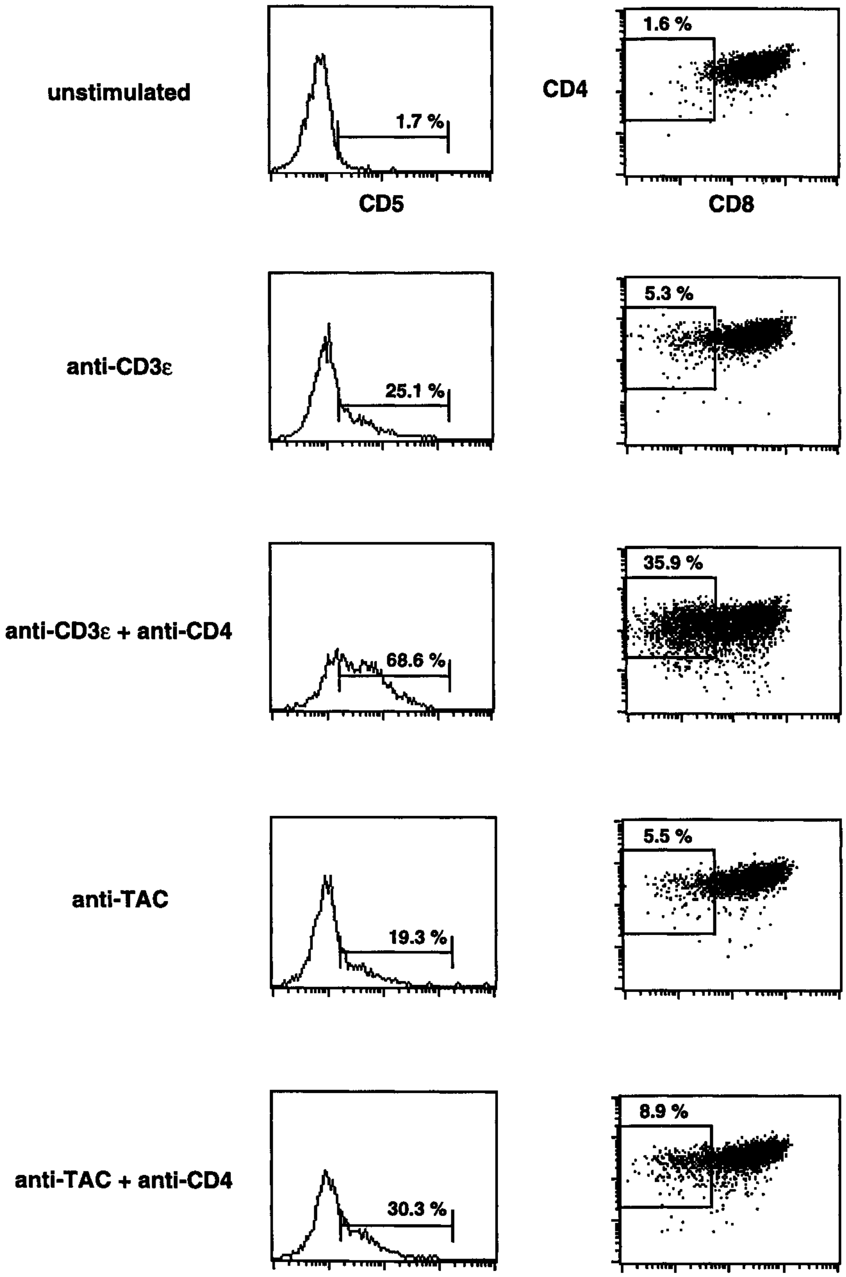

FIGURE 6 Comparison of CD3 and TTζ-mediated differentiation. ZPDPK cells were stimulated with anti-CD3ε or anti-Tac mAb in the presence or absence of coimmobilized anti-CD4 mAb as indicated. Cells were harvested after 3 days of culture and analyzed as in Fig. 1.

that the ζ chain cannot function as an autonomous module but rather requires interaction with other molecules of the CD3 complex for efficient signaling during immature T-cell differentiation.

The specific deficit in TTζ-mediated signaling that limits its ability to induce the differentiation of DPK cells is not known, but we have found a reduced ability of this chimeric protein to mediate proximal signaling events. Early studies looking at tyrosine phosphorylation of intracellular substrates in transfected T-cell lines led to contrasting reports as to whether there are different or identical patterns of tyrosine phosphorylation of intracellular substrates induced on crosslinking of the endogenous TCR/CD3 or chimeric ε or ζ protein alone (Irving and Weiss, 1991; Letourneur and Klausner, 1992; Aoe et al., 1994; Shinkai et al., 1995). We found that stimulation of ZPDPK cells via the chimeric TTζ protein results in tyrosine phosphorylation of a subset of the intracellular substrates that are phosphorylated by anti-CD3 stimulation. The phosphorylation of some substrates was also markedly reduced. Thus, there may be both quantitative and qualitative differences in downstream signals. This is in contrast to the results of Shinkai et al. (1995), where identical patterns of phosphorylation were induced by crosslinking of either endogenous CD3ε or the chimeric TTζ chain in transgenic mouse thymocytes. It is possible that these disparate findings result from the transformed state of DPK cells. It is also possible, however, that these differences reflect the different outcomes of anti-CD3 mediated crosslinking in thymocytes (apoptosis) versus DPK cells (differentiation). In this regard, recent evidence suggests that the ras signaling pathway is required for positive but not negative selection (Alberola-Ila et al., 1995; Swan et al., 1995). We also found that the differentiation of DPK cells is dependent on the p21ras signaling pathway (manuscript in preparation). Thus, one possibility is that the TTζ chimeric protein is poorly coupled to the ras signaling cascade and is unable to mediate differentiation of DPK cells while still able to deliver the signals necessary to elicit cell death of normal thymocytes.

Examination of cytokine production as indicative of late cellular activation led to the following observations. In response to a primary stimulus of anti-CD3ε antibody, undifferentiated DPK cells produce low levels of IL-2, whereas differentiated DPK cells secrete predominantly IL-4. In contrast, ZPDPK cells stimulated by crosslinking of the TTζ chain produced no detectable IL-2. This finding was somewhat surprising because chimeric ζ-mediated stimulation of the IL-2 gene has been reported in T-cell hybridomas (Irving and Weiss, 1991; Letourneur and Weiss, 1992; Aoe et al., 1994). However, as seen for normal mature T cells, the activation state of the T cell can have a profound effect on the ability of ζ-chain chimeras to mediate activation (Brocker and Karjalainen, 1995). Although Shinkai et al. (1995) have reported that ζ-chain signaling can elicit proliferative responses in single-positive thymocytes comparable to signaling through the intact TCR, Brocker and Karjalainen (1995) have reported that mature T cells must be previously activated before an isolated ζ-chain module can function. Our results similarly suggest that the developmental state of the T cell may effect the ability of isolated TCR-signaling components to function. Following differentiation, ZPDPK cells were found to produce only low levels of IL-4 in response to anti-Tac mAb, again consistent with a qualitative or quantitative deficit in TTζ-mediated signaling.

On DPK differentiation, there is often a greater percentage of DPK cells that express CD5 than those that exhibit a $CD8^{lo/-}$ phenotype (Figs. 1 and 6). This may reflect a lower threshold for the induction of CD5 than for downregulation of CD8 gene expression. This is consistent with the finding that anti-CD3ε mAb can induce CD5 surface expression much more efficiently than full differentiation of DPK cells. We have also previously shown that thymic epithelial cells induce loss of CD8 but only low-level expression of CD69 on DPK cells (Poirier et al., 1994). Thus, there may be subtle differences in the signals necessary to effect changes in gene expression of each of these differentiation markers. In contrast to the results with CD3 crosslinking, TTζ-mediated differentiation was not significantly enhanced by coimmobilization with anti-CD4 mAb. This may reflect a qualitative difference in CD3- and TTζ-generated signals, as suggested by differences in the patterns of tyrosine phosphorylation of cellular substrates. Alternatively, the chimeric protein external domain may fail to interact appropriately with CD4 and thus induce only partial signals.

It is clear from these experiments that TCR stimulation alone is a poor substitute for the normal cellular interactions that mediate immature T-cell differentiation. The critical question for the future is what are the relevant costimulatory molecules and signals given by thymic epithelial cells that regulate and enhance this process. We are currently exploiting this system to address this issue.

### MATERIALS AND METHODS

### Cell Lines and Reagents

The isolation and characterization of the murine $CD4^+$ $8^+$ T-cell line, DPK, has been previously described (Kaye and Ellenberger, 1992). All DPK cell lines were maintained in EHAA medium (Irvine Scientific, Santa Ana, California), supplemented with 10% FCS (GIBCO, Grand Island, New York), 2 mM glutamine, 50 μM 2-mercaptoethanol, 100 U/ml penicillin, and 100 mg/ml streptomycin. The transfected fibroblast antigen-presenting cell line, DCEK-ICAM (Kuhlman et al., 1991), that expresses $E^k$ class II MHC and ICAM-1 was maintained in RPMI medium (Whittaker, Walkersville, Maryland) supplemented as before with the addition of mycophenolic acid, xanthine, hypoxanthine, and G418.

### Expression of TTζ Chimera in DPK Cells

The TTζ construct encodes a chimeric molecule composed of the human CD25 extracellular and transmembrane domains and the murine ζ-chain intracellular domain (Letourneur and Klausner, 1991). This construct was cloned into a retroviral vector encoding puromycin resistance (Morgenstern and Land, 1990), and DPK cells were infected with recombinant retrovirus as described previously (Miller et al., 1993). Puromycin-resistant DPK cells were subsequently sorted on a FACStar (Becton Dickinson, Mountain View, California) for expression of human CD25.

### Antibodies and Flow Cytometric Analysis

Analysis of cell-surface markers was performed using the following antibodies: anti-Tac (hCD25) (Pharmingen, San Diego, California), phycoerythrin-conjugated anti-CD4 (Gibco-BRL, Grand Island, New York), Red613-conjugated anti-CD8 (Gibco-BRL), biotinylated anti-CD69 (Pharmingen), and biotinylated anti-CD5 (Pharmingen). DPK cells were stained as previously described (Kaye and Ellenberger, 1992) and data analyzed on a FACScan using CellQuest software (Becton Dickinson, Mountain View, California). Dead cells were gated out of the analysis based on light scatter.

### Primary Stimulation and Differentiation Assays

Tissue-culture plates were coated with either anti-CD3ε mAb 145-2C11 (Leo et al., 1984) or anti-Tac mAb (hCD25) (Immunotech, Westbrook, Maine) (20 μg/ml, unless indicated otherwise) in PBS, in the presence or absence of 10 μg/ml anti-CD4 mAb (Pharmingen) overnight at 4°C, followed by multiple washes with PBS prior to addition of DPK cells. Cells were plated at $2 \times 10^5$ cells/0.5 ml per well in a 24-well plate, harvested after 3 days of culture, and three-color stained for FACS analysis, as described before.

### Restimulation Assays for Determination of IL-2/IL-4 Production

Pigeon cytochrome c carboxy-terminal peptide (PC), residues 88–104, was synthesized at the Scripps Research Institute. DCEK-ICAM cells were plated at $1 \times 10^5$ cells/well in 24-well plates approximately 16 hours prior to addition of $3 \times 10^5$ DPK, in the presence or absence of 1 μM PC. After 24 hr of culture, DPK cells were transferred to a fresh plate and 0.5 ml fresh medium added. At 48 hr, cells were again transferred with additional media containing recombinant human IL-2 in a final concentration of 100 U/ml to enhance survival (Kaye and Ellenberger, 1992). Cultures were harvested on day 4, three-color stained with biotinylated CD5 (Pharmingen), anti-CD4-PE (Gibco-BRL), and anti-CD8-613 (Gibco-BRL), and analyzed on a FACScan. Cells were washed and resuspended at $4 \times 10^5$ cells/ml and plated at 0.5 ml/well in a 48-well tissue-culture plate previously coated with either 2C11 antibody or anti-Tac (hCD25), as described before. Twenty-four hours later supernatants were harvested and tested for the presence of cytokine by use of the IL-2/IL-4-responsive NK cell line, as previously described (Kaye and Ellenberger, 1992). Supernatants were added at 5% v/v per well in triplicate. In some assays, an anti-IL-

4 blocking mAb (BVD4; Pharmingen) was added at 8 μg/ml final concentration.

### Anti-Phosphotyrosine Immunoblotting

Cells were washed and resuspended at 2 × $10^7$/ml in culture medium and prewarmed in a 37°C water bath. Cells were stimulated with polystyrene latex beads (5.2 μm in diameter; Interfacial Dynamics Corp., Portland, Oregon) previously conjugated with either rat immunoglobulin, as a negative control, 2C11 or anti-Tac (hCD25) antibody, resuspended in culture medium at 2 ×$10^7$/ml and prewarmed as before (Taddie et al., 1994). One-tenth milliliter of antibody-conjugated beads were mixed with 0.1 ml of appropriate cells, pelleted briefly in a microcentrifuge, and then incubated at 37°C for 30 min. Following stimulation, tubes were placed on ice, 1 ml of ice cold PBS + 0.1 mM $Na_3VO_4$ was added, and the cells pelleted again. Cell pellets were solubilized in lysis buffer (PBS containing 1% NP-40/1% SDS/2mM Na3VO4/1mM NaF/1mM PMSF/5 μg/ml aprotinin/5 μg/ml leupeptin) on ice for 30 min. The detergent-insoluble material was removed by centrifugation at 14,000 × g for 10 min, and lysates mixed with SDS sample buffer containing 2-mercaptoethanol, then boiled for 5 min prior to gel electrophoresis. Approximately 1/3 of the total lysate was resolved on a 12% polyacrylamide gel alongside prestained molecular-weight markers (Biorad, Hercules, California) and transferred onto PVDF membrane at 25 V overnight. The blot was blocked with 2% BSA/Tris-buffered saline-Tween (TBS-T; 10 mM Tris/150 mM NaC/0.05% Tween 20, pH 8.0) for 2 hr at room temperature with rocking, washed twice in TBS-T, then probed with anti-phosphotyrosine antibody 4G10 (UpState Biotechnology Inc., Lake Placid, New York) in 2% BSA/TBS-T for 2 hr at room temperature with rocking. Following extensive washing, the blot was developed with goat anti-mouse IgG-HRPO (BioRad) in 2% BSA/TBS-T for 1 hr at room temperature with rocking, washed, and developed with ECL reagents (Amsersham, Arlington Heights, Illinois).

### Intracellular $Ca^{2+}$ Assay

Cells were stained with either 2C11 or anti-Tac (hCD25) mAb at 10 μg/ml in 4% FCS/PBS for 30 min at room temperature. After washing, cells were incubated with 10 μg/ml Indo-1 (Molecular Probes, Junction City, Oregon) in 10% BSA/Indo-loading buffer (Taddie et al., 1994) for 30 min at 32°C, washed, and resuspended in 0.1% BSA/Indo-loading buffer at 3 × $10^5$ cells/ml. The cells were stimulated by preincubating at 37°C for 10 min prior to crosslinking with secondary antibodies at a final concentration of 10 μg/ml rabbit anti-hamster IgG or goat anti-rat IgG (Caltag, San Francisco). Results are presented as the ratio of FL4/FL5 fluorescent intensity over time as measured by a FACSvantage flow cytometer (Becton Dickinson). Ionomycin was obtained from Calbiochem (La Jolla, California).

### ACKNOWLEDGMENTS

The authors thank Dr. Richard Klausner for the generous gift of the TTζ chimera construct and Dr. Anne O'Rourke for thoughtful advice and technical assistance. We also want to acknowledge the contributions of Joe Trotter for advice and assistance with the calcium mobilization assay and Carina Torres for assistance in cell sorting.

This work was supported by grants from the National Institutes of Health (AI31231 and AI33219). J.D. was supported by NIH training grant HL07195. This is manuscript 9752-IMM from the Scripps Research Institute.

### *References*

Alberola-Ila, J., Forbush, K. A., Seger, R., Krebs, E. G. and Perlmutter, R. M. (1995) Selective requirement for MAP kinase activation in thymocyte differentiation. *Nature*, **373**, 620.

Aoe, T., Goto, S., Ohno, H. and Saito, T. (1994) Different cytoplasmic structure of the CD3ζ family dimer modulates the activation signal and function of T cells. *Int. Immunol.*, **6**, 1671.

Baldari, C. T., Di Somma, M. M., Milia, E., Bergman, M. and Telford, J. L. (1995) Interactions between the tyrosine kinases p561ck, p59fyn and p50csk in CD4 signaling in T cells. *Eur. J. Immunol.*, **25**, 919.

Brocker, T. and Karjalainen, K. (1995) Signals through T cell receptor-ζ chain alone are insufficient to prime resting T lymphocytes. *J. Exp. Med.*, **181**, 1653.

Burkhardt, A. L., Stealey, B., Rowley, R. B., Mahajan, S., Prendergast, M., Fargnoli, J. and Bolen, J. B. (1994) Temporal regulation of non-transmembrane protein tyrosine kinase enzyme activity following T cell antigen receptor engagement. *J. Biol. Chem.*, **269**, 23642.

Chu, K. and Littman, D. R. (1994) Requirement for kinase activity of CD4-associated $p56^{lck}$ in antibody-triggered T cell signal transduction. *J. Biol. Chem.*, **269**, 24095.

Danielian, S., Alcover, A., Polissard, L., Stefanescu, M., Acuto, O., Fischer, S. and Fagard, R. (1992) Both T cell receptor (TcR)-CD3 complex and CD2 increase the tyrosine kinase activity of p56lck: CD2 can mediate TcR-CD3-independent and CD45-dependent activation of p56lck. *Eur. J. Immunol.*, **22**, 2915.

DeKoning, J., Carbone, F. R. and Kaye, J. (1995) Contrast between class I and class II MHC-mediated differentiation of a CD4 + CD8 + T cell line: implications for lineage commitment. *Intl. Immunol.*, **7**, 541.

Dutz, J. P., Ong, C. J., Marth, J. and Teh, H.-S. (1995) Distinct differentiative stages of CD4 + CD8 + thymocyte development defined by the lack of coreceptor binding in positive selection. *J. Immunol.*, **154**, 2588.

Flaswinkel, H., Barner, M. and Reth, M. (1995) The tyrosine activation motif as a target of protein tyrosine kinases and SH2 domains. *Semin. Immunol.*, **7**, 21.

Gelfand, E. W., Cheung, R. K., Mills, G. B. and Grinstein, S. (1988) Uptake of extracellular Ca2 + and not recruitment from internal stores is essential for T lymphocyte proliferation. *Eur. J. Immunol.*, **18**, 917.

Goldsmith, M. A. and Weiss, A. (1988) Early signal transduction by the antigen receptor without commitment to T cell activation. *Science*, **240**, 1029.

Irving, B. A., Chan, A. C. and Weiss, A. (1993) Functional characterization of a signal transducing motif present in the T cell antigen receptor ζ chain. *J. Exp. Med.*, **177**, 1093.

Irving, B. A. and Weiss, A. (1991) The cytoplasmic domain of the T cell receptor ζ chain is sufficient to couple to receptor-associated signal transduction. *Cell*, **64**, 891.

Kaye, J. and Ellenberger, D. L. (1992) Differentiation of an immature T cell line: a model of thymic positive selection. *Cell*, **71**, 1.

Kearse, K. P., Takahama, Y., Punt, J. A., Sharrow, S. O. and Singer, A. (1995) Early molecular events induced by T cell receptor (TCR) signaling in immature CD4 + CD8 + thymocytes: Increased synthesis of TCR-α protein is an early response to TCR signaling that compensates for TCR-α instability, improves TCR assembly, and parallels other indicators of positive selection. *J. Exp. Med.*, **181**, 193.

Kuhlman, P., Moy, V. T., Lollo, B. A. and Brian, A. A. (1991) The accessory function of murine intercellular adhesion molecule-1 in T lymphocyte activation. *J. Immunol.*, **146**, 1773.

Ledbetter, J. A., Gilliland, L. K. and Schieven G. L. (1990) The interaction of CD4 with CD3/Ti regulates tyrosine phosphorylation of substrates during T cell activation. *Semin. Immunol.*, **2**, 99.

Leo, O., Foo, M., Sachs, D. H., Samelson, L. E. and Bluestone, J. A. (1984) Identification of a monoclonal antibody specific for a murine T3 polypeptide. *Proc. Natl. Acad. Sci. USA*, **84**, 1374.

Letourner, F. and Klausner, R. D. (1991) T-cell and basophil activation through the cytoplasmic tail of T-cell-receptor ζ family proteins. *Proc. Natl. Acad. Sci. USA*, **88**, 8905.

Letourneur, F. and Klausner, R. D. (1992) Activation of T cells by a tyrosine kinase activation domain in the cytoplasmic tail of CD3ε. *Science*, **255**, 79.

Liu, C. P., Ueda, R., She, J., Sancho, J., Wand, B., Weddell, G., Loring, J., Kurahara, C., Dudley, E. C., Hayda, A., Terhorst, C. and Huang, M. (1993) Abnormal T cell development in CD3ζ–/– mutant mice and identification of a novel T cell population in the intestine. *EMBO J.*, 12, 4863.

Love, P. E., Shores, E. W., Lee, E. J., Grinberg, A., Munitz, T. I., Westphal, H. and Singer, A. (1994) Differential effects of ζ and ε transgenes on early α/β T cell development. *J. Exp. Med.*, 179, 1485.

Love, P. E., Shores, E. W., Johnson, M. D., Tremblay, M. L., Lee, E. J., Grinberg, A., Huang, S. P., Singer, A. and Westphal, H. (1993) T cell development in mice that lack the z chain of the T cell antigen receptor complex. *Science*, 261; 918.

Malissen, M., Gillet, A., Rocha, B., Trucy, J., Vivier, E., Boyer, C., Kontgen, F., Brun, N., Mazza, G., Spanopoulou, E., Guy-Grand, D. and Malissen, B. (1993) T cell development in mice lacking the CD3z/h gene. *EMBO J.*, 12, 4347.

Miceli, M. C. and Parnes, J. R. (1993) Role of CD4 and CD8 in T cell activation and differentiation. *Adv. Immunol.*, 53, 59.

Miller, D. A., Miller, D. G., Garcia, J. V. and Lynch, C. M. (1993) Use of retroviral vectors for gene transfer and expression. *Meth. Enzymol.*, 217, 581.

Morgenstern, J. P. and Land, H. (1990) Advanced mammalian gene transfer: high titre retroviral vectors with multiple drug selection markers and a complementary helper-free packaging cell line. *Nucleic Acids Res.*, 18, 3587.

O'Rourke, A. M. and Mescher, M. F. (1994) Signals for activation of CD8-dependent adhesion and costimulation in CTLs. *J. Immunol.*, 152, 4358.

Ohno, M., Aoe, T., Taki, S., Kitamura, D., Ishida, Y., Rajewsky, K. and Saito, T. (1993) Developmental and functional impairment of T cells in mice lacking CD3ζ chains. *EMBO J.*, 12, 4357.

Poirier, G., Lo, D., Reilly, C. and Kaye, J. (1994) Discrimination between thymic epithelial cells and peripheral antigen presenting cells in the induction of immature T cell differentiation. *Immunity*, 1, 385.

Rahemtulla, A., Fung-Leung, W. P., Schilham, M. W., Kundig, T. M., Sambhara, S. R., Narendran, A., Arabian, A., Wakeham, A., Paige, C. J., Zinkernagel, R. M., Miller, R. G. and Mak, T. W. (1991) Normal development and function of CD8 *T* cells but markedly decreased helper cell activity in mice lacking CD4. *Nature*, 353, 180.

Reth, M. (1989) Antigen receptor tail clue. *Nature*, **338**, 383.

Romeo, C., Amiot, M. and Seed, B. (1992) Sequence requirements for induction of cytolysis by the T cell antigen/Fc receptor ζ chain. *Cell*, **68**, 889.

Romeo, C. and Seed, B. (1991) Cellular immunity to HIV activated by CD4 fused to T cell or Fc receptor polypeptides. *Cell*, **64**, 1037.

Shi, Y., Sahai, B. M. and Green, D. G. (1989) Cyclosporin A inhibits activation-induced cell death in T-cell hybridomas and thymocytes. *Nature*, **339**, 625.

Shinkai, Y., Ma, A., Cheng, H. L. and Alt, F. W. (1995). CD3ε and CD3ζ cytoplasmic domains can independently generate signals for T cell development and function. *Immunity*, **2**, 401.

Shores, E. W., Huang, K., Tran, T., Lee, E., Grinberg, A. and Love, P. E. (1994) Role of TCR ζ chain in T cell development and selection. *Science*, **266**, 1047.

Smith, C. A., Williams, G. T., Kingston, R., Jenkinson, E. T. and Owen, J. J. T. (1989) Antibodies to CD3/T-cell receptor complex induce death by apoptosis in immature T cells in thymic cultures. *Nature*, **337**, 181.

Sussman, J. J., Bonifacino, J. S., Lippincott-Schwartz, J., Weissman, A. M., Saito, T., Klausner, R. D. and Ashwell, J. D. (1988) Failure to synthesize the T cell CD3-$\zeta$ chain: Structure and function of a partial T cell receptor complex. *Cell*, **52**, 85.

Swan, K. A., Alberola-Ila, J., Gross, J. A., Appleby, M. W., Forbush, K. A., Thomas, J. F. and Perlmutter, R. M. (1995). Involvement of $p21^{ras}$ distinguishes positive and negative selection in thymocytes. *EMBO J.*, **14**, 276.

Taddie, J. A., Hurley, T. R., Hardwick, B. X. and Sefton, B. M. (1994) Activation of B- and T-cells by the cytoplasmic domain of the B-cell antigen receptor proteins Ig-$\alpha$ Ig-$\beta$. *J. Biol. Chem.*, **269**, 13529.

Tarakhovsky, A., Kanner, S. B., Hombach, J., Ledbetter, J. A., Muller, W., Killeen, N. and Rajewsky, K. (1995) A role for CD5 in TCR-mediated signal transduction and thymocyte selection. *Science*, **269**, 535.

Tsygankov, A. Y., Broker, B. M., Fargnoli, J., Ledbetter, J. A. and Bolen, J. B. (1992) Activation of tyrosine kinase p60fyn following T cell antigen receptor cross-linking. *J. Biol. Chem.*, **267**, 18259.

Wegener, A. M. K., Letourneur, F., Hoeveler, A., Brocker, T., Luton, F. and Malissen, B. (1992) The T cell receptor/CD3 complex is composed of at least two autonomous transduction modules. *Cell*, **68**, 83.

Weiss, A. (1991) Molecular and genetic insights into T cell antigen receptor structure and function. *Annu. Rev. Genet.*, **25**, 487.

Weissman, A. M., Baniyash, M., Hou, D., Samelson, L. E., Burgess, W. H. and Klausner, R. D. (1988) Molecular cloning of the zeta chain of the T cell antigen receptor. *Science*, **239**, 1018.

